# The Value of ^18^F-FDG PET/CT Mathematical Prediction Model in Diagnosis of Solitary Pulmonary Nodules

**DOI:** 10.1155/2018/9453967

**Published:** 2018-03-28

**Authors:** Ling Wang, Yao Chen, Kun Tang, Jie Lin, Hong Zhang

**Affiliations:** ^1^Department of Nuclear Medicine, The Second Affiliated Hospital of Zhejiang University School of Medicine, Hangzhou, China; ^2^Department of Nuclear Medicine, The First Affiliated Hospital of Wenzhou Medical University, Wenzhou, China; ^3^Zhejiang University Medical PET Center, Hangzhou, China; ^4^Institute of Nuclear Medicine and Molecular Imaging of Zhejiang University, Hangzhou, China; ^5^Key Laboratory of Medical Molecular Imaging of Zhejiang Province, Hangzhou, China; ^6^Department of PET/CT, Radiology Imaging Center, The First Affiliated Hospital of Wenzhou Medical University, Wenzhou, China

## Abstract

**Purpose:**

To establish an ^18^F-fluorodeoxyglucose (^18^F-FDG) positron emission tomography/computed tomography (PET/CT) mathematical prediction model to improve the diagnosis of solitary pulmonary nodules (SPNs).

**Materials and Methods:**

We retrospectively reviewed 177 consecutive patients who underwent ^18^F-FDG PET/CT for evaluation of SPNs. The mathematical model was established by logistic regression analysis. The diagnostic capabilities of the model were calculated, and the areas under the receiver operating characteristic curve (AUC) were compared with Mayo and VA model.

**Results:**

The mathematical model was *y* = exp⁡(*x*)/[1 + exp⁡(*x*)], *x* = −7.363 + 0.079 × age + 1.900 × lobulation + 1.024 × vascular convergence + 1.530 × pleural retraction + 0.359 × the maximum of standardized uptake value (SUV_max_). When the cut-off value was set at 0.56, the sensitivity, specificity, and accuracy of our model were 86.55%, 74.14%, and 81.4%, respectively. The area under the receiver operating characteristic curve (AUC) of our model was 0.903 (95% confidence interval (CI): 0.860 to 0.946). The AUC of our model was greater than that of the Mayo model, the VA model, and PET (*P* < 0.05) and has no difference with that of PET/CT (*P* > 0.05).

**Conclusion:**

The mathematical predictive model has high accuracy in estimating the malignant probability of patients with SPNs.

## 1. Introduction

Solitary pulmonary nodules (SPNs) refer to a round or oval lung lesion, with clear margins, no more than 30 mm in the maximum diameter, completely surrounded by healthy lung parenchyma, and not associated with satellite lesions, atelectasis, pneumonia or hilar enlargement, and mediastinal lymph nodes [1]. With the widespread use of multidetector CT technology, the detection rate of SPNs has been increased [2]. It is reported that more than 150,000 new cases of SPN are detected annually in the United States [3]. Although most SPNs are benign, about 35% are primary malignancies [4], most of them in TNM stage IA, with a 5-year survival rate for patients of 61% to 75% [5]. Because of the small lesion volume and the lack of specific CT imaging features between benign and malignant lesions, estimating the probability of malignancy is a common problem [6]. In addition, because it is difficult to identify SPNs, about half of patients with lung cancer miss the optimal timing of surgery, resulting in a 10% to 15% decreased 5-year survival rate [7]. Thus, improving the accuracy of SPNs diagnosis is therefore critical to the treatment options and prognosis for patients.

PET measuring the degree of glucose uptake of tissue has proven to be an excellent modality for tumor imaging [8]. Positron emission tomography/computed tomography (PET/CT) displays both CT morphologic features of the lesions and metabolic information at the molecular level. The value of ^18^F-fluorodeoxyglucose (^18^F-FDG) PET/CT in the diagnosis of SPNs has been widely recognized [9, 10]. However, the accuracy of interpretation of PET/CT for SPNs depended on the radiologist's personal experience, the artifacts, and quantitative errors caused by the CT attenuation for PET, and the variance within and/or between observers may lead to misinterpretation of PET/CT results [2].

Mathematical model is an objective evaluation method based on statistics; therefore, it is expected to provide a reliable and accurate SPN diagnosis. Most of the previously reported SPN mathematical predictive models developed to overcome subjectivity were established on the basis of clinical features, such as the widely cited Mayo model [11] and VA model [12].

To our knowledge, few models include metabolic parameters based on ^18^F-FDG PET/CT imaging as predictors, and the value of such model in diagnosing SPN was still unconfirmed. Therefore, the purpose of this study was to establish a mathematical prediction model including glucose metabolism as one of the predictors and compare the diagnostic value of this model with the Mayo model, the VA model, PET/CT, and PET to identify the usefulness of this model.

## 2. Materials and Methods

### 2.1. Materials

The local ethics committee and the institutional review board approved this retrospective study. Informed consent was signed for all patients in the study. From October 2011 to September 2013, 228 consecutive cases, who were confirmed with SPNs and had undergone PET/CT, were retrospectively analyzed. All malignant nodules were confirmed by histopathologic examination of the tissue obtained by surgery or biopsy. All benign nodules were confirmed by pathologic diagnosis or clinical follow-up. Clinical follow-up included a significant reduction in lesion or complete regression of anti-inflammatory or antituberculosis treatment. When SPN is clinically and radiologically stable for at least 2 years, a definitive benign diagnosis is established. Patients with the longest diameter of SPNs < 7 mm, a history of primary lung cancer, or related thoracic surgery with distant metastasis were excluded. Fifty-one participants were excluded on the basis of the above clinical criteria.

Nineteen clinical characteristics of patients were collected, including gender, age, smoking history, time since quitting, history of cancer, family history of cancer, lesion diameter, position, CT value, border, ground-glass opacity, lobulation, vascular convergence, pleural retraction, spiculation, calcification, vacuoles, cavitation, and the maximum of standardized uptake value (SUV_max_).

### 2.2. PET/CT Image Acquisition

All patients fasted for at least 6 hours (blood glucose < 110 mg/dl) before PET/CT acquisition. PET/CT scans were performed 60 min after injection of 3.7 MBq/Kg FDG using a whole-body PET/CT scanner (Gemini TF 64, Philips, Netherlands). The CT scan parameters were reconstructed of 64 (detectors) × 0.625 mm (detector collimation) with a tube voltage of 120 kV, a tube current of 50 mA, a pitch of 0.829, and a rotation time of 0.5 seconds. The reconstruction thickness and intervals were 5 mm. The data were reconstructed using a 512 × 512 pixel matrix. Following CT scan, a three-dimensional mode was used to obtain PET images from the base of the skull to the middle of the thigh. The emission scan time for each bed position was 1.5 min. PET images were reconstructed with CT attenuation correction using the ordered subsets expectation maximization. All collected data were transferred to the Philips EBW 3.0 workstation to reconstruct the images of transverse, coronal, sagittal PET, CT, and PET/CT fusion images.

### 2.3. Image Analysis

#### 2.3.1. CT Image Interpretation

CT imaging features of the nodules were independently analyzed by two radiologists with 11 and 30 years of experience, respectively, who did not know the pathologic findings and metabolic activity of lesions before this visual assessment. The size, density, position, boundary, ground-glass opacity, lobulation, vascular convergence, pleural retraction, spiculation, calcification, vacuole, and cavity were analyzed. The maximum diameter of the nodule was measured using CT, and nodule density was measured by the average CT value. The nodule's position was divided into right upper lobe, right middle lobe, right lower lobe, left upper lobe, and left lower lobe. The presence or absence of the other nine CT features is represented as “1” or “0,” respectively.

#### 2.3.2. PET Image Interpretation

The metabolic characteristics of PET images were interpreted in consensus by two experienced nuclear medicine physicians (both with 13 years of experience) who were unaware of the patient's history and CT findings. SUV was measured in SPNs using the region of interest (ROI) technique. Using 40% of the maximum SUV value of lesions as the threshold, the lesion ROI and the SUV_max_ of SPNs lesions were outlined automatically. If there was decreased uptake or no uptake of SPNs, ROI could not be outlined automatically. Using early axial CT image as a reference, the ROI of lesions on the transaxial slices was manually sketched. SUV_max_ was defined as the highest activity of the lesion.

#### 2.3.3. PET/CT Image Interpretation

PET/CT images were interpreted as benign or malignant according to the following criteria: If PET and CT diagnoses were concordant, the diagnosis was determined to be benign or malignant. If the two were discordant, lesions were diagnosed by the following criteria: If the signs of benign and malignant lesions are typical in CT, regardless of the metabolic characteristics, morphologic diagnosis is the priority; if lesions had typical high FDG metabolic signs, PET diagnosis has priority; if both PET and CT signs were not typical, we combined the lesion morphology with metabolism to make the determination. Diagnosis was made by 5-point Likert scale: 0, definitely benign; 1, more likely benign; 2, probably benign; 3, probably malignant; 4, more likely malignant; and 5, definitely malignant [13].

### 2.4. Statistical Analysis

All data were analyzed using the Statistical Package for the Social Sciences, version 22.0. A *P* value < 0.05 was considered statistically significant. The results of benign and malignant lesions were used as dependent variables, while the nineteen clinical characteristics of patients are used as independent variables; univariate and multivariate logistic regression analysis were then performed. The diagnostic sensitivity, specificity, accuracy, positive predictive value, and negative predictive value of SPNs diagnosis were calculated. The difference in AUC between PET/CT and other methods was tested with the *χ*^2^ statistic.

## 3. Results

### 3.1. Clinical Data of SPNs

**Figure 1:**
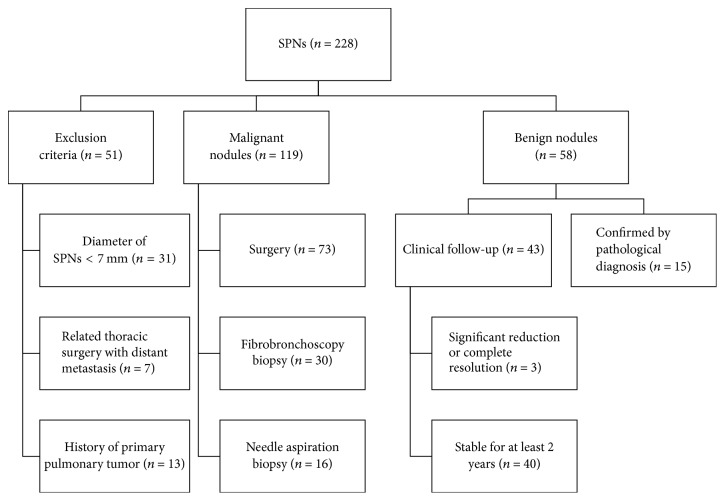
The flowchart of the recruitment for SPNs and the process of determination of the diagnosis for all SPNs enrolled.

One hundred seventy-seven patients (95 male, 82 female; age range, 26 to 85 years; mean age, 61.59 ± 10.53 years) met the recruitment criteria. Recruitment rules for the study are shown in Figure 1. The mean nodule diameter was 18.89 ± 7.02 mm (range, 6 to 30 mm). Of these patients, 119 malignant nodules were pathologically confirmed, and 58 benign nodules were confirmed by pathology or clinical follow-up. The final diagnosis of SPNs and subtypes are listed in Table 1. The results of nineteen clinical characteristics of patients were shown in Table 2.

### 3.2. SPN Diagnosis by PET/CT

**Figure 2:**
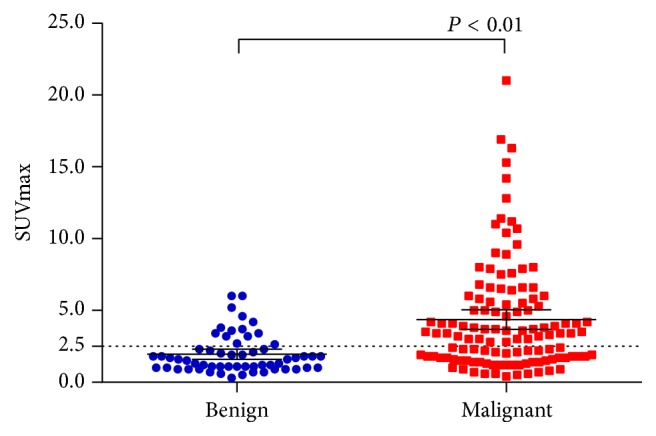
Distribution of SUV_max_ in benign and malignant SPNs. Notice the overlaps of SUV_max_ between the two groups.

**Figure 3:**
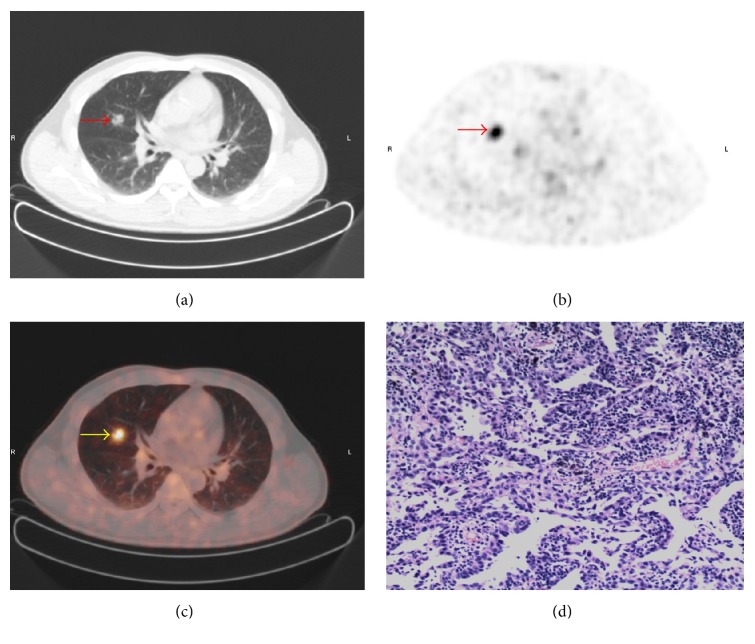
A typical PET/CT finding in a 54-year-old male patient. (a) On CT image, the nodule in the right middle lobe is observed (arrow); the maximum diameter of nodule is 16 mm. (b) On PET and (c) on PET/CT image, increased ^18^F-FDG uptake is detected; SUV_max_ = 3.9. (d) Pathologic examination suggests gland squamous cell carcinoma. The *y* value calculated by our model is 0.73. The result of our model is consistent with PET/CT and pathological results.

**Figure 4:**
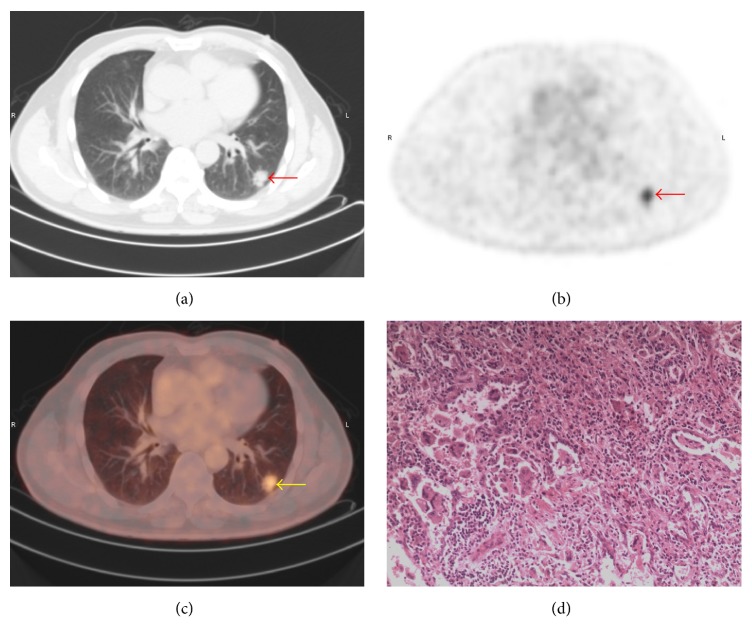
A false-positive PET/CT finding in a 55-year-old male patient. (a) On CT image, the nodule in the left lower lobe with lobulation, spiculation, and vacuole sign is observed (arrow); the maximum diameter of nodule is 18 mm. (b) On PET and (c) on PET/CT image, increased ^18^F-FDG uptake is detected; SUV_max_ = 2.2. (d) Pathologic examination suggests cryptococcal infection. The *y* value calculated by our model is 0.91. The result of our model is consistent with PET/CT.

**Figure 5:**
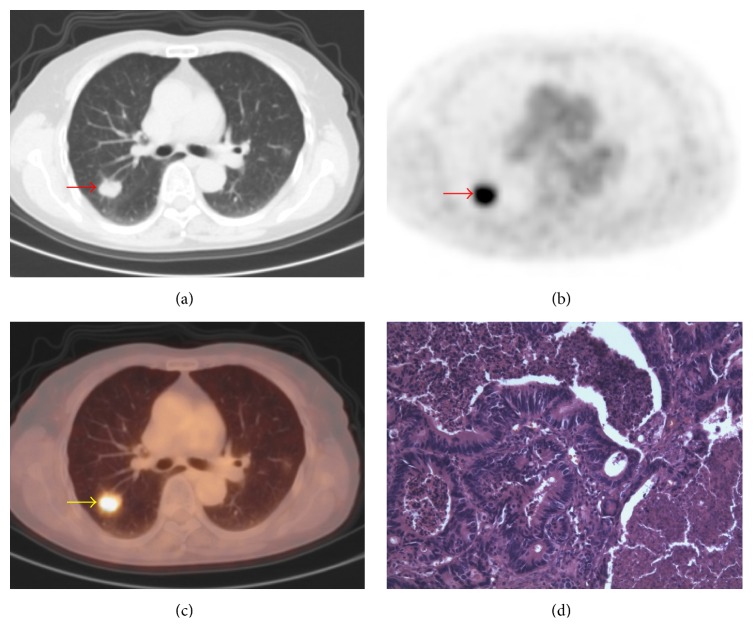
A false-negative PET/CT finding in a 64-year-old female patient. (a) On CT image, the nodule in the right upper lobe with lobulation and low density is observed (arrow); the maximum diameter of nodule is 17 mm. (b) On PET and (c) on PET/CT image, increased ^18^F-FDG uptake is detected; SUV_max_ = 7.4; the result of PET imaging is infection. (d) Pathologic examination suggests metastatic adenocarcinoma. The *y* value calculated by our model is 0.73. The result of our model is consistent with PET and pathological result but inconsistent with PET/CT.

**Figure 6:**
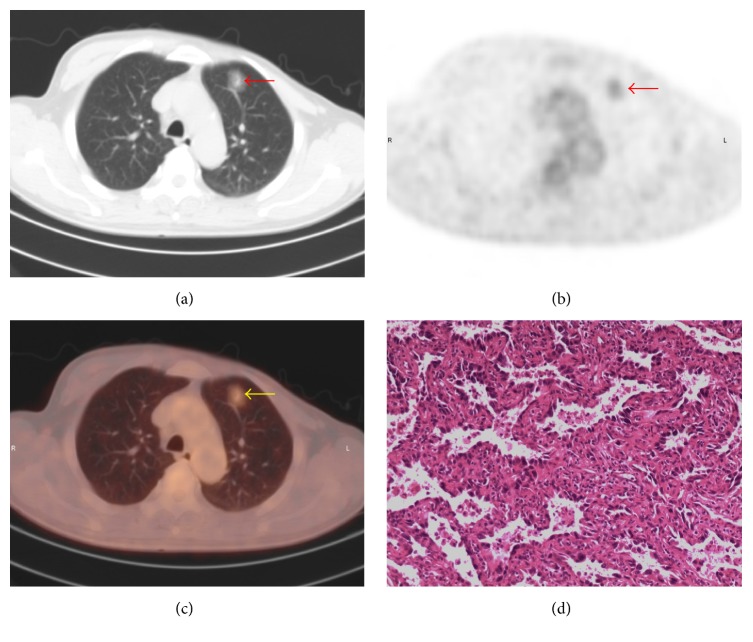
A typical PET/CT finding in a 56-year-old male patient. (a) On CT image, the nodule in the left upper lobe with high density in the center and low density in the surrounding tissue and a clear border is observed (arrow); the maximum diameter of nodule is 22 mm. (b) On PET and (c) on PET/CT image, SUV_max_ = 1.7. (d) Pathologic examination suggests bronchioloalveolar carcinoma. The *y* value calculated by our model is 0.09. The result of our model is inconsistent with PET/CT and pathologic result.

There are overlaps of SUV_max_ between benign and malignant SPNs. The SUV_max_ distribution of benign and malignant nodules is shown in Figure 2. Point ≥ 3 is used as standard to diagnose malignant SPNs. The sensitivity, specificity, and accuracy of PET/CT in diagnosing SPNs were 98.32%, 77.59%, and 91.50%, respectively. Typical cases of PET/CT imaging are shown in Figures 3, 4, 5, and 6.

### 3.3. Univariate and Multivariate Analysis

The univariate analysis results are shown in Table 2. There were significant differences in age, lesion diameter, CT value, lobulation, pleural retraction, vascular convergence, spiculation, vacuoles, and SUV_max_ values between benign and malignant SPNs. By multivariate logistic regression analysis, patient age (*P* value = 0.001), lobulation (*P* value = 0.000), vascular convergence (*P* value = 0.043), pleural retraction (*P* value = 0.001), and SUV_max_ (*P* value = 0.006) were identified as independent predictors for the diagnosis of benign and malignant SPNs, which are shown in Table 3.

### 3.4. Model Establishment and Interpretation

Multivariate logistic regression analysis was used to establish the mathematical model for ^18^F-FDG PET/CT diagnosis of SPNs. The result is as follows: *y* = exp⁡(*x*)/[1 + exp⁡(*x*)], *x* = −7.363 + 0.079 × age + 1.90 × lobulation + 1.024 × vascular convergence + 1.530 × pleural retraction + 0.359 × SUV_max_, where *e* is the natural logarithm; age in years, the presence or absence of lobulation, vascular convergence, and pleural retraction were recorded as “1” or “0.” The *y* value of 0.56 was set as the cut-off point according to the ROC analysis, when *P* < 0.56 should be considered as benign disease, while *P* > 0.56 should be considered as malignant SPNs. The sensitivity, specificity, and accuracy of the mathematical model to predict benign and malignant SPNs were 86.55%, 74.14%, and 81.4%, respectively. For the diagnosis of SPNs, there are 129 malignant and 48 benign nodules determined by PET/CT, 126 malignant and 51 benign nodules determined by our model, 125 malignant and 52 benign nodules determined by Mayo model, and 136 malignant and 41 benign nodules determined by PET.  Table 4 lists the details.

Most of the results interpreted by our model are consistent with PET/CT. For example, in a 54-year-old male patient, the result of our model was identical to PET/CT and consistent with pathologic findings (Figure 3). And in another 55-year-old male patient, the result of our model is malignancy which was also identical to PET/CT but inconsistent with pathologic findings. Both the results of our model and PET/CT are false-positive in this patient (Figure 4). However, there are some inconsistencies between the results interpreted by our model and PET/CT. For example, in a 64-year-old female patient with metastatic adenocarcinoma, the diagnosis of our model was malignancy but the diagnosis of PET/CT was infection which is false-negative (Figure 5). It is noticeable that in patients without those morphological features described in our model, the diagnoses of which tend to be false-negative when interpreted by our model, which is inconsistent with PET/CT (Figure 6).

The Mayo model [12] was defined as *p* = exp⁡(*x*)/[1 + exp⁡(*x*)], *x* = −6.8272 + (0.0391 × age) + (0.7917 × smoking history) + (1.3388 × cancer history) + (0.1274 × diameter) + (1.0407 × speculation) + 0.7838 × the upper lobe.

The VA model [13] was defined as *p* = exp⁡(*x*)/[1 + exp⁡(*x*)], *x* = −8.404 + (2.061 × smoke) + (0.779 × age 10) + (0.112 × diameter) − (0.567 × years quit 10).

### 3.5. ROC (Receiver Operating Characteristic) Curve Analysis

**Figure 7:**
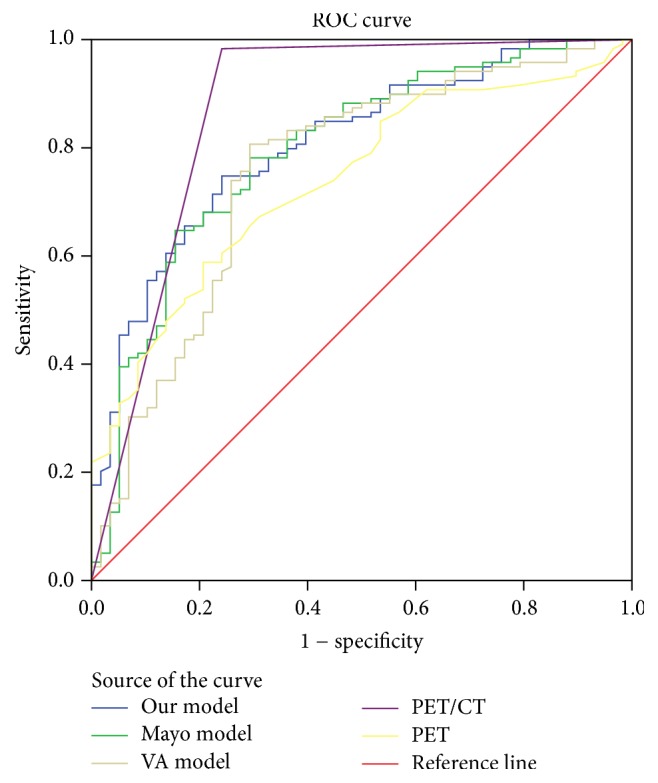
ROC curves for our model, Mayo model, VA model, PET/CT, and PET.

The ROC curve plotted by the mathematical prediction model, PET/CT, PET, and Mayo model for diagnosis of SPNs were drawn. The AUCs were calculated, as shown in Figure 7. By *Z* test, the AUC was compared. The AUCs of PET/CT and our model have no significant difference. Both AUCs of PET/CT and our model are significantly larger than that of SUV_max_ (Table 5).

## 4. Discussion

The American College Of Chest Physicians Lung Cancer Guidelines suggest that individuals with pulmonary nodules should be evaluated by estimating the probability of malignancy either by using clinical judgment or by using a validated model to guide management of SPNs [14]. Many mathematical models have been developed [11, 12, 15]. Many researchers used quantitative models to evaluate benign and malignant SPNs, such as the Mayo Clinic model and VA model [11, 12]. The Mayo model is one of the most widely used ones. The clinical characteristics such as age, smoking history, and cancer history and morphologic features such as SPN diameter, speculation, and the upper lobe of the position are defined as predictors of malignancy by the reported models. However, Shinohara et al. [16] stated that the Mayo model yielded unsatisfactory results in differentiating malignant from benign SPNs and reported that the model performance tended to be weak in predicting malignancies. Furthermore, a recent study applied the Mayo model in a work including 288 consecutive cases of SPNs demonstrated that the Mayo model proved to be of limited value in SPN assessment [17]. The Mayo model should be used with caution when it is used in the preoperative diagnosis of SPNs. Our study applied the information of 177 patients with SPNs to the Mayo model and showed that the model's AUC was 0.79 (95% confidence interval (CI): 0.72 to 0.86), which is in line with the reported literature.

FDG uptake is a measurement of glucose metabolism and is used to distinguish benign from malignant nodules. Gould et al. [18] had done meta-analysis that included 40 SPNs ^18^F-FDG PET studies and stated that the sensitivity and specificity of the diagnosis of ^18^F-FDG PET for benign and malignant SPNs were 96.8% and 77.8%, respectively. PET/CT is a molecular imaging modality that combines anatomic, morphologic characteristics of CT with metabolic information of PET to obtain accurate diagnosis of SPNs, the diagnostic performance of which is greater than that by CT or PET alone [19]. We found that the AUC of PET/CT to diagnose malignant SPNs is 0.95 (95% CI: 0.92 to 0.98), and the sensitivity, specificity, and accuracy of PET/CT at diagnosing SPNs were 98.32%, 77.59%, and 91.50%, respectively, which is in line with previous research. However, PET/CT diagnosis of SPNs has limitations. Quite a few benign nodules share almost the same morphologic and metabolic characteristics with malignant lesions, thus potentially leading to false-positive findings. Moreover, some malignant SPNs with low-grade FDG uptake contribute to false-negative results. In addition, accuracy of PET/CT diagnosis depends on the physician's clinical experience, which may not be reproducible.

Therefore, to develop a useful mathematical model to characterize SPNs is paramount and is this study's purpose. Age [12], pleural retraction [11], tumor diameter [20], speculation [21], calcification [15], border [15], and the upper lobe [22] were reported as independent factors for the judgment of benign and malignant SPNs. In this study, a total of 19 independent factors were included in univariate and multivariate analysis, and logistic regression equation was then established. It is notable that not only clinical characteristics and morphologic features were analyzed as independent variables, but also FDG uptake calculated as the SUV_max_. Finally, our model includes five predictors, namely, age, lobulation, pleural retraction, vascular convergence, and SUV_max_. Of these predictors, lobulation has the highest diagnostic odds ratio, indicating higher diagnostic specificity of this clinical sign.

Our model's distinguishing aspect is that it includes SUV_max_ as one of the predictors. The diagnostic sensitivity, specificity, and accuracy of malignant SPNs predicted by our model were 86.55%, 74.14%, and 81.4%, respectively, and 67.23%, 68.97%, and 67.21% for PET, respectively. Our model showed better performance (AUC is 0.90, 95% CI: 0.86 to 0.95) than PET alone (AUC is 0.74, 95% CI: 0.6 to 0.81) for the diagnosis of SPNs. In this study, the bronchioloalveolar carcinoma with a lower level of FDG uptake than other types of lung cancer occupies a large proportion of malignant nodules, and the FDG uptake is increased in some benign nodules such as tuberculosis, inflammatory pseudotumor, and pulmonary cryptococcosis; this may contribute to the results that there are overlaps of SUV_max_ values between benign and malignant nodules, which may be the reason that the diagnostic odds ratio for SUV_max_ is 0.359 in our model.

The population characteristics of our model were different from the Mayo model and VA model. Most patients recruited in the Mayo Clinic model were current or former smokers. However, in this study, only 47 patients with SPNs were smokers and very few of them quit smoking before this examination. According to the Mayo model, smoking history is reported to be an independent factor in the diagnosis of malignant SPNs [11]. In addition, the VA model [12] stated that time since smoking cessation was an independent factor for SPN malignancies. However, the parameters such as smoking history and time since smoking cessation were not identified as predictors in our model after regression analysis. This is the other advantage of our model, that it could be applied in patients without smoking history, which means that it is more versatile than the Mayo model and VA model.

Univariate analysis showed that nodule size and vacuole have statistically significant differences between the benign and malignant nodules, but nodule size and vacuole were not defined as independent predictors when calculated by multivariate analysis. We also noted a high proportion of malignant nodules (67.23%) in the recruited cases, which may be due to selection bias of many benign nodules diagnosed by other routine examinations and did not undergo PET/CT examination; therefore, they were not included in this work.

There is no statistically significant difference between the AUC of PET/CT and our model, indicating a similarly high efficacy of these two methods in diagnosing SPNs. Although most of the diagnosis interpreted by our model is consistent with PET/CT, there are still some differences between our model and PET/CT for those patients with no obvious CT features. Our study had several limitations. The establishment of our model was based on a retrospective study, and the sample size was still not large enough. Because the establishment and validation of the model are based on the same patient group, the model needs to be further validated by other groups of patients with SPNs.

## 5. Conclusion

In this retrospective study of 177 patients with SPNs, we established a mathematical regression model to assess the probability of SPN malignancies. Our model has the same diagnostic value as PET/CT in predicting the malignancy of SPNs, the results of which were interpreted by experienced radiologists. The result interpreted by our model is more accurate than those by the Mayo model, the VA model, and PET alone, and it is more versatile than the Mayo model and VA model.

## Figures and Tables

**Table 1:** SPNs: final diagnosis and subtypes.

Diagnosis	*n*	%
Benign nodules	58	32.77%
Tuberculosis	7	3.95%
Interstitial pneumonia	4	2.26%
Inflammatory pseudotumor	2	1.13%
Hamartoma	8	4.52%
Atypical adenomatous hyperplasia	2	1.13%
Giant lymph node hyperplasia	1	0.56%
Organizing pneumonia	2	1.13%
Benign pulmonary tumor	9	5.08%
Cryptococcosis	2	1.13%
Bronchial cysts	1	0.56%
Nonspecific inflammation	20	11.30%
Malignant nodules	119	67.23%
Adenocarcinoma	50	28.25%
Squamous cell carcinoma	16	9.04%
Adenosquamous carcinoma	5	2.82%
Bronchial alveolar carcinoma	18	10.17%
Mucinous adenocarcinoma	2	1.13%
Other	28	15.82%

**Table 2:** Nineteen clinical characteristics of patients with SPNs.

	Benign	Malignant	*P* value
Gender			
Male	32	63	0.781
Female	26	56	
Age, years	56.19 ± 10.82	64.22 ± 9.36	0.000
Diameter, mm	16.00 ± 6.67	20.34 ± 6.19	0.000
Smoking history	11	36	0.112
Time since quitting, years	0.69 ± 0.48	0.84 ± 0.26	0.778
History of cancer	2	11	0.062
Family history of cancer	3	8	0.062
Position			
Right upper lobe	21	38	0.663
Right middle lobe	5	9	
Right lower lobe	11	22	
Left upper lobe	9	31	
Left lower lobe	12	19	
CT value	−71.6 ± 143.24	−43.69 ± 101.99	0.138
Border	22	57	0.213
Ground glass opacity	7	18	0.586
Lobulation	38	108	0.000
Vascular convergence	9	74	0.000
Pleural retraction	9	70	0.000
Cavity	5	4	0.136
Spiculation	34	105	0.000
Calcification	2	6	0.634
Vacuole	5	37	0.001
SUV_max_	1.95 ± 1.34	4.37 ± 3.81	0.000

**Table 3:** The five predictors of the logistic regression mathematical model for SPNs.

Factor	Regression coefficient	*P* value	95% CI	
			Lower	Upper
Age	0.079	0.001	1.033	1.134
Lobulation	1.900	0.000	2.485	17.987
Vascular Convergence	1.024	0.043	1.032	7.512
Plural retraction	1.530	0.001	1.840	11.592
SUV_max_	0.359	0.006	1.106	1.855
Constant	−7.363	0.000		

**Table 4:** The results of SPNs diagnosed by our model, Mayo model, VA model, PET/CT, and PET.

Methods	Diagnosis	Final diagnosis	
		Malignant	Benign
Our model	Malignant	106	20
	Benign	13	38
Mayo model	Malignant	100	25
	Benign	19	33
VA model	Malignant	105	32
	Benign	14	26
PET/CT	Malignant	116	13
	Benign	3	45
PET	Malignant	103	33
	Benign	16	25

**Table 5:** The AUCs for our model, the Mayo model, the VA model, PET/CT, and PET.

	Area	Standard error	Significance	95% CI		*Z* value	*P* value
				Lower	Upper		
Our model	0.903	0.022	0.000	0.860	0.946	-	-
Mayo model	0.789	0.038	0.000	0.715	0.864	2.60^a^	<0.05
VA model	0.746	0.042	0.000	0.665	0.828	3.31^b^	>0.05
PET/CT	0.953	0.015	0.000	0.924	0.983	−1.88^c^	<0.05
PET	0.738	0.038	0.000	0.664	0.812	3.76^d^	<0.05

*Z* value: ^a^our model versus Mayo model; ^b^our model versus VA model; ^c^our model versus PET/CT; ^d^our model versus PET.
